# Medical Diagnosis

**Published:** 1898-09

**Authors:** 


**Medical Diagnosis.** By J. J. Graham Brown, M.D. Fourth Edition. Pp. xx., 428. Edinburgh: William F. Clay. 1897.

As this book has reached a fourth edition, it has evidently attained success. In the preface the author states that this edition has been thoroughly revised, and in great measure re-written, especially as regards the chapters dealing with the examination of the gastric contents, of the blood and of the urine, and that relating to the nervous system.

So many books of the same kind have been published within the last two or three years, and many of them excellent in every way, that it is difficult for the student to make a selection. The merit of this particular work lies chiefly in the excellent opening chapter on the general conditions of the patient, and those dealing with the investigation of physical signs, of the urine, and of the nervous system. The sections devoted to the description and *rationale* of physical signs of the heart and lungs are especially good, and are clearly written. That on the examination of the blood is rather too short, and the author would have done better either to have given more space to the ophthalmoscopic appearances of diseases of the optic nerve, or to have contented himself with a description of the use of the ophthalmoscope and of the normal fundus oculi. The chapter on cerebral and mental functions requires considerable expansion to make it of practical value, and in that on aphasia some instructions should have been given as to the mode of examining patients suffering from the different forms of aphasia. Still, to keep a book of this kind within anything like moderate limits, there is necessarily much difficulty in selecting what must be dealt with and what left out or very briefly treated; and on the whole the author has dealt with his matter with much judgment, and his statements are sound, and written in a scientific spirit. The illustrations are good, and the book is well printed.

